# Effects of 4-Hexylresorcinol on Protein Expressions in RAW 264.7 Cells as Determined by Immunoprecipitation High Performance Liquid Chromatography

**DOI:** 10.1038/s41598-019-38946-4

**Published:** 2019-03-04

**Authors:** Min Keun Kim, Cheol Soo Yoon, Seong Gon Kim, Young Wook Park, Sang Shin Lee, Suk Keun Lee

**Affiliations:** 1 Department of Oral and Maxillofacial Surgery, College of Dentistry, Gangneung-Wonju National University, and Institute of Oral Science, Gangneung, Korea; 2 Department of Oral Pathology, College of Dentistry, Gangneung-Wonju National University, and Institute of Oral Science, Gangneung, Korea

## Abstract

4-Hexylresorcinol (4HR) is a small organic compound that is used as an additive antiseptic and antioxidant, but its molecular properties have not been clearly elucidated. The present study explored the cellular effects of 4HR on RAW 264.7 cells by immunoprecipitation high-performance liquid chromatography (IP-HPLC) using 216 antisera. 4HR-treated cells showed significant decreases in the expressions of proliferation-related proteins, cMyc/MAX/MAD network, p53/Rb/E2F and Wnt/β-catenin signalings, epigenetic modifications, and protein translation. Furthermore, 4HR suppressed the expressions of growth factors and proteins associated with RAS signaling, NFkB signaling, inflammation, and osteogenesis, but elevated the expressions of proteins associated with p53-mediated and FAS-mediated apoptosis, T-cell immunity, angiogenesis, antioxidant, and oncogenic signaling. In a 4HR adherence assay, TNFα, PKC, osteopontin, and GADD45 were strongly adherent to 4HR-coated beads, whereas IL-6, c-caspase 3, CDK4, and c-caspase 9 were not. Many 4HR adherent proteins were expressed at lower levels in 4HR treated RAW 264.7 cells than in non-treated controls, whereas 4HR non-adherent proteins were expressed at higher levels. These observations suggest 4HR affects the expressions of proteins in an adhesion-dependent manner and that its effects on proteins are characteristic and global in RAW 264.7 cells.

## Introduction

4-Hexylresorcinol (4HR) is a substituted phenol synthesized from resorcinol and caproic acid^1^. It is used as an antimicrobial in toothpastes and skin lotions^2^, and as a preservative for fresh fruits and vegetables^3^. 4HR has bactericidal^4^, anthelmintic^5^ and potential antineoplastic activities^6^, and thus, is also used as an antiseptic in mouthwashes and skin wound cleansers^7^. 4HR may also inhibit oxidative DNA damage by enhancing the activities of antioxidant enzymes, including glutathione peroxidase and glutathione reductase, which facilitate the scavenging of ROS (reactive oxygen species) by glutathione (GSH)^8^, and thus, it is also used to prevent the enzymatic browning of shrimps and different fruits^9^.

Immunoprecipitation high-performance liquid chromatography (IP-HPLC) had been used previously by several authors to detect organic compounds including peptides quantitatively, but the technique used was complicated and of limited applicability^10,11^. Recently, a new IP-HPLC protocol was developed to determine protein expression levels in different biological fluids, such as blood serum, urine, saliva^12^, inflammatory exudates^13–15^, cancer tissues^16^, and coffee extract^17^. IP-HPLC is comparable to enzyme-linked immunosorbent assay (ELISA), but the former uses protein A/G agarose beads in buffer solution and UV spectroscopy to determine protein concentrations, whereas the latter uses fluorescence-conjugated antibodies fixed in plastic wells and fluoroscopy. Furthermore, multiple trials have shown that IP-HPLC can be used to rapidly determine multiple protein levels accurately (±5% standard deviation) and reproducibly.

The human body is believed to be relatively tolerant to 4HR, which is now being increasingly used as a food additive and antiseptic agent, and 4HR has been suggested to have anti-inflammatory^18^, anticancer^19^, and angiogenesis^20^ effects. However, its molecular interactions and signaling in cells are not well understood and its biochemical properties remain ambiguous. Thus, the present study was undertaken to investigate and compare the cellular effects of 4HR, and to elucidate the molecular mechanism responsible for the effect of 4HR in RAW 264.7 cells using IP-HPLC.

## Results

### Summary of workflow

4HR was applied to RAW 264.7 cell cultures for 8, 16, or 24 hours, and protein samples were subjected to IP-HPLC using 216 antisera. It was found that 4HR differentially influenced the expressions of many essential proteins, and thus, 4HR adherence assays were performed to investigate interactions between 4HR and proteins. 4HR adhered to the surfaces of acrylamide beads in 50 mM Tris buffer (pH 7.5) and treated with protein extract of RAW 264.7 cells. Eluted protein mixtures were examined by IP-HPLC, and protein expressional changes were compared with the 4HR binding efficiencies with different proteins to determine whether proteins that interacted with 4HR were upregulated or downregulated in RAW 264.7 cells. IP-HPLC results were plotted as line graphs versus culture time and those obtained after 16 hours of culture (when protein expressional changes were greatest) were plotted as circular graphs.

### Effects of 4HR on the expressions of proliferation-related proteins in RAW 264.7 cells

**Figure 1:**
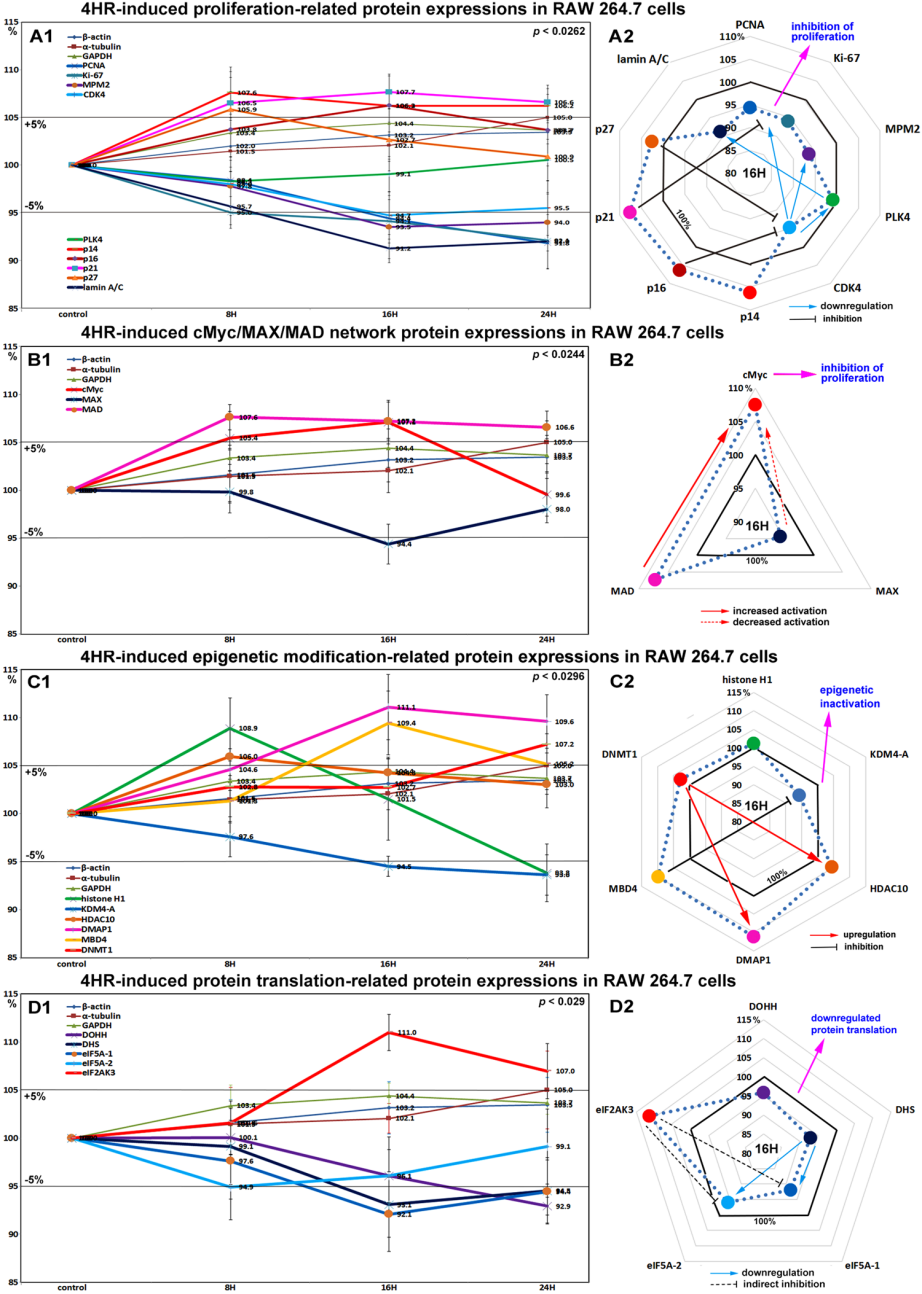
Expressions of proliferation-related proteins (**A1** and **A2**), cMyc/MAX/MAD network proteins (**B1** or **B2**), epigenetic modification proteins (**C1** and **C2**), and protein translation-related proteins (**D1** or **D2**) in 4HR-treated RAW 264.7 cells as determined by IP-HPLC. Line graphs, (**A1**, **B1**, **C1**, and **D**1) show protein expressional changes in the same scale (%) with respect to culture time (8, 16, or 24 hours), whereas circular graphs (**A2**, **B2**, **C2**, and **D2**) show protein expressions after 16 hours of culture.

RAW 264.7 cells treated with 4HR for 8, 16, or 24 hours exhibited gradual decreases in the levels of proliferation-activating proteins [Ki-67 by 7.9%, proliferation cell nuclear antigen (PCNA) by 8.2%, lamin A/C by 8.8%, mitotic protein M2 (MPM2) by 6.5%, and cyclin dependent kinase 4 (CDK4) by 5.3%], but increases in the levels of proliferation-inhibiting proteins [p14 by 7.6%, p16 6.3%, p21 7.7%, and p27 by 5.9%] versus non-treated controls. These expressional changes became noticeable after 16 and 24 hours of 4HR treatment (Fig. 1A1 and A2), but remained at <±10%. On the other hand, the expression of polo-like kinase (PLK4) tended to decrease as did those of housekeeping proteins. These results suggest cell proliferation was slightly inhibited by 4HR treatment due to its effects on proliferation-inhibiting proteins, therefore, we considered 4HR might have an anti-proliferative effect that is enough to delay or inactivate mitosis.

### Effects of 4HR on the expressions of cMyc/MAX/MAD network proteins in RAW 264.7 cells

4HR increased the expressions of cMyc and MAD by 7.1 and 7.2% at 16 hours, respectively, but reduced the expression of MAX by 5.6% at 16 hours versus non-treated controls. MAD expression increased by a maximum of 7.6% after 8 hours of 4HR treatment and this was maintained until 24 hours, and cMyc expression increased by a maximum of 7.1% at 16 hours but decreased to the control level at 24 hours (Fig. 1B1 and B2). These expressional changes of the cMyc/MAX/MAD network co-occurred inhibition of proliferation by 4HR (Fig. 1A1 and A2).

### Effects of 4HR on the expressions of epigenetic modification-related proteins in RAW 264.7 cells

Histone H1 and histone deacetylase 10 (HDAC10) expressions increased to 108.9% and 106% of those in non-treated controls after 8 hours of 4HR treatment, respectively, but then gradually decreased to 93.8% and 103% at 24 hours, respectively. The expression of lysine-specific demethylase 4D (KDM4D) was reduced by 6.4% at 24 hours, while that of DNA methyltransferase 1-associated protein 1 (DMAP1) and was gradually increased by 11.1% at 16 hours and maintained at 109.6% at 24 hours, DNA (cytosine-5)-methyltransferase 1 (DNMT1) expression was increased by 7.2% at 24 hours, and also methyl-CpG binding domain 4 (MBD4) expression was increased by 9.4% at 16 hours and by 5.2% at 24 hours (Fig. 1C1 and C2). These results suggest 4HR might inactivate DNA transcription in RAW 264.7 cells, and that this epigenetic effect of 4HR might be related to the downregulations of proliferation-related proteins.

### Effects of 4HR on the expressions of translation-related proteins in RAW 264.7 cells

RAW 264.7 cells treated with 4HR showed gradual reductions in protein translation-related protein levels versus non-treated controls. Deoxyhypusine hydroxylase (DOHH) expression reduced by 3.9% and 7.1% at 16 and 24 hours, respectively, and deoxyhypusine synthase (DHS) expression was reduced by 6.9% and 5.5% at 16 and 24 hours, respectively. The protein expressions of objective factors of protein translation, that is, eukaryotic translation initiation factor 5A-1 (eIF5A-1) and eIF5A-2 proteins were also reduced by 7.9% and 3.9% at 16 hours, respectively, while that of eukaryotic translation initiation factor 2-α kinase 3 (eIF2AK3; an inactivator of eIF2) was increased by 11% at 16 hours (Fig. 1D1 and D2). It was thought that the rapid reduction of the expressions of translation-related proteins by 4HR might induce global inactivation of cellular signaling, although these changes in protein levels tended to disappear after 24 hours of 4HR treatment.

### Effects of 4HR on the expressions of p53/Rb/E2F signaling proteins in RAW 264.7 cells

**Figure 2:**
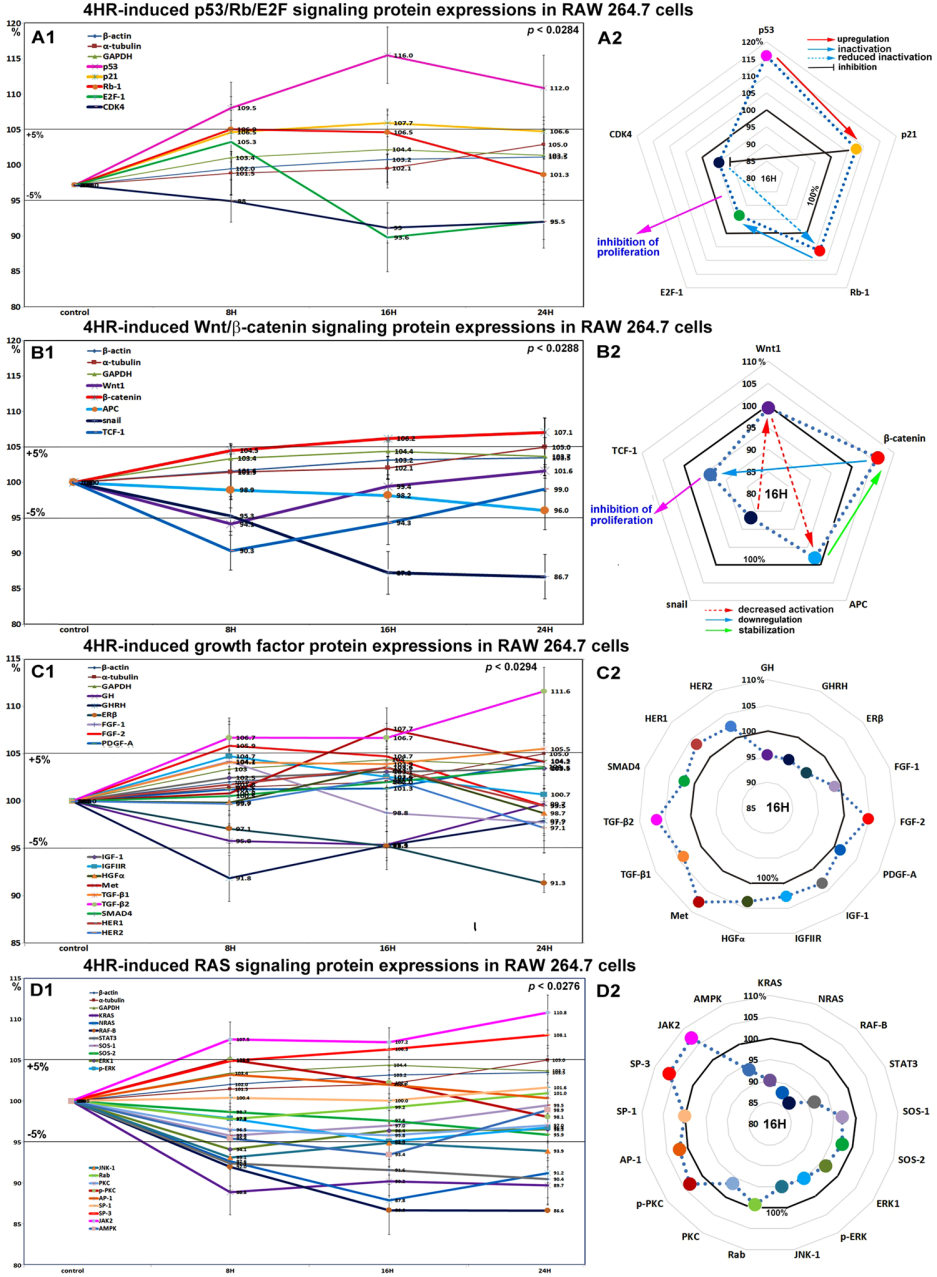
Expressions of p53/Rb/E2F signaling proteins (**A1** and **A2**), Wnt/β-catenin signaling proteins (**B1** or **B2**), growth factors (**C1** and **C2**), and RAS signaling proteins (**D1** or **D2**) in RAW 264.7 cells treated with 4HR, as determined by IP-HPLC. Line graphs, (**A1**, **B1**, **C1**, and **D1**) show protein expressional changes in the same scale (%) with respect to culture time (8, 16, or 24 hours), whereas circular graphs (**A2**, **B2**, **C2**, and **D2**) showed protein expression levels after 16 hours of culture.

4HR increased the expression of p53 in RAW 264.7 cells by 16% at 16 hours and by 12% at 24 hours versus non-treated controls. Rb-1 expression was also slightly increased by 6.9% and 6.5% at 8 and 16 hours, respectively, but then decreased to the control level (1.3% increase) at 24 hours. Notably, p21 expression increased by 7.7% at 16 hours, whereas CDK4 expression gradually decreased by 5.3% and 4.5% at 16 and 24 hours, respectively. The expression of the objective transcription factor E2F-1 decreased by 6.4% at 16 hours and by 4.5% at 24 hours (Fig. 2A1 and A2). We supposed these protein expression changes in p53/Rb/E2F signaling, increases in the expressions of p53, Rb-1, and p21, but concurrent decreases in the expressions of CDK4 and E2F could contribute to 4HR-induced reductions in proliferation.

### Effects of 4HR on the expressions of Wnt/β-catenin signaling proteins in RAW 264.7 cells

β-Catenin expression in RAW 264.7 cells was increased by 7.1% by 4HR at 24 hours versus non-treated controls, but the expressions of snail, Wnt1, and adenomatous polyposis coli (APC) were reduced by 13.3%, 5.9%, and 4%, respectively. The expression of the transcription factor, T-cell factor 1 (TCF-1), was moderately decreased by 9.7% at 8 hours and by 5.7% at 16 hours (Fig. 2B1 and B2). These downregulations of Wnt/β-catenin signaling by 4HR might also contribute to the reduced RAW 264.7 cell proliferation caused by 4HR.

### Effects of 4HR on the expressions of growth factor-related proteins in RAW 264.7 cells

RAW 264.7 cells treated with 4HR showed gradual decreases in the expressions of growth hormone (GH), growth hormone-releasing hormone (GHRH), platelet-derived growth factor-A (PDGF-A), fibroblast growth factor-1 (FGF-1), and estrogen receptor β (ERβ) by 2.9–8.7% during 24 hours of treatment versus non-treated controls, while the expressions of transforming growth factor-β1 (TGF-β1), TGF-β2, SMAD4 (mothers against decapentaplegic homolog 4), FGF-2, and Met were increased by 5.5%, 11.6%, 3.5%, 5.9%, and 7.7%, respectively. The expressions of other growth factor-related proteins, including HGFα, insulin-like growth factor-1 (IGF-1), IGFIIR, HER1, and HER2, like those of housekeeping proteins changed minimally (by ±5%), however, the expressions of many growth factors and related proteins tended to increase slightly (Fig. 2C1 and C2). These results indicate 4HR alters the expressions of growth factors required for the growth and regeneration of RAW 264.7 cells, but does so negatively for GH, GHRH, PDGF-A, FGF-1, and ERβ, and positively for TGF-β1, TGF-β2, SMAD4, FGF-2, and Met.

### Effects of 4HR on the expressions of RAS signaling proteins in RAW 264.7 cells

4HR gradually suppressed the expressions of RAS signaling proteins in RAW 264.7 cells. Most RAS signaling proteins were downregulated after 4HR treatment versus non-treated controls. The expressions of KRAS, NRAS, RAF-B, and STAT3 (signal transducer and activator of transcription-3) were moderately decreased by 10.3%, 12.2%, 13.4%, and 9.6%, respectively, at 16 to 24 hours. Similarly, the expressions of protein kinase C (PKC), AMP-activated protein kinase (AMPK), Jun N-terminal protein kinase-1 (JNK-1), extracellular signal–regulated kinase 1 (ERK1), and p-ERK were slightly decreased by 3.5%, 6.6%, 6.1%, 3.6%, and 3.2%, respectively. The expressions of SOS-1 (son of sevenless homolog-1), SOS-2, and specificity protein-1 (SP-1) decreased by <5% over 24 hours, while the expressions of p-PKC, and activating protein-1 (AP-1) increased slightly by 5.5% and 3.2% at 8 hours, but then gradually decreased to 99.6% and 100.3% at 24 hours, respectively (Fig. 2D1 and D2). These results indicate major signaling for cellular growth and protection, that is, RAS signaling, was greatly inhibited by 4HR. On the other hand, the expression of SP3, a bifunctional transcription factor that either stimulates or represses the transcriptions of numerous genes gradually increased by 8.1% at 24 hours, and the expression of JAK2 (a non-receptor tyrosine kinase implicated in signaling by members of the type II cytokine receptor family) also increased by 10.8%.

### Effects of 4HR on the expressions of NFkB signaling proteins in RAW 264.7 cells

**Figure 3:**
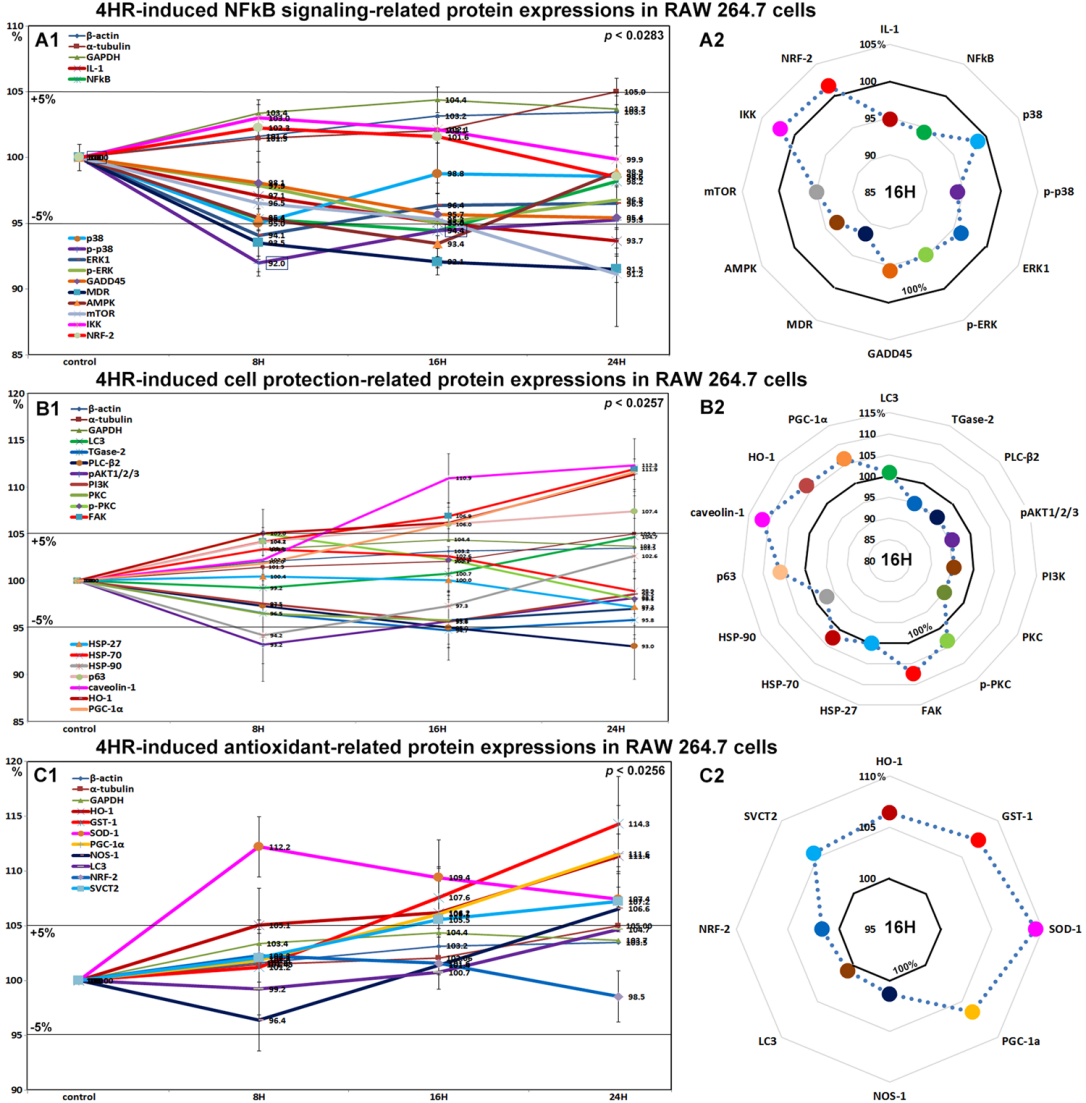
Expressional changes in NFkB signaling proteins (**A1** and **A2**), cell protection-related proteins (**B1** or **B2**), and antioxidant-related proteins (**C1** and **C2**) induced by 4HR in RAW 264.7 cells, as determined by IP-HPLC. Line graphs, (**A1**, **B1**, and **C1**) show protein expressional changes in the same scale (%) with respect to culture time (8, 16, or 24 hours), while circular graphs (**A2**, **B2**, and **C2**) showed protein expression levels after 16 hours of culture.

4HR gradually decreased the expressions of NFkB signaling proteins in RAW 264.7 cells. The expression of NFkB (nuclear factor kappa-light-chain-enhancer of activated B) was decreased by 5.6% at 16 hours versus non-treated controls, while the expressions of IKK (ikappaB kinase) and NRF2 (nuclear factor (erythroid-derived 2)-like 2) were slightly increased by 3% and 2.3% at 8 hours, respectively. Most proteins that activate NFkB signaling were downregulated by 4HR, that is, p38 by 5% at 8 hours, p-p38 by 8% at 8 hours, MDR (multi-drug resistance) by 9.5% at 24 hours, mTOR (mammalian target of rapamycin) by 8.8% at 24 hours, IL-1 by 6.3% at 24 hours, and GADD45 by 4.6% at 24 hours. Proteins that inhibit NFkB signaling were also downregulated by 4HR, that is, AMPK by 6.6% at 16 hours and 1.1% at 24 hours, ERK1 by 5.9% at 8 hours, and p-ERK by 5% at 16 hours (Fig. 3A1 and A2). These results indicate major aspects of NFkB signaling were greatly inhibited by 4HR, but its effects were broad and not selective for NFkB signaling pathways.

### Effects of 4HR on the expressions of cell protection-related proteins in RAW 264.7 cells

The expressions of cell protection-related proteins in RAW 264.7 cells were reduced by 4HR; pAKT1/2/3 by 6.8% at 8 hours, PLC-β2 (1-phosphatidylinositol-4,5-bisphosphate phosphodiesterase β-2) by 7% at 24 hours, PI3K (phosphatidylinositol-3-kinase) by 4.6% at 16 hours, and PKC (protein kinase C) by 4.2% at 16 hours versus non-treated controls. p-PKC expression was slightly increased by 5% at 8 hours but gradually decreased to 98.1% in 24 hours, and the expression of its downstream signaling component FAK was also increased by 6.9% at 16 hours and by 11.9% at 24 hours. The expression of the cellular chaperone protein HSP-70 (heat shock protein-70), increased by 3.3% at 8 hours but gradually decreased to 98.9% at 24 hours. HSP-27 expression slightly decreased to 97.2% at 24 hours, and HSP-90 expression decreased to 94.2% at 8 hours but then gradually increased to 102.6% at 24 hours. The expression of PGC-1α (master regulator of mitochondrial biogenesis) increased by 6% at 16 hours and by 11.8% at 24 hours, and that of its downstream target protein, HO**-**1 (heme oxygenase**-**1**)** was increased by 6.2% at 16 hours and by 11.4% at 24 hours. In addition, the expression of caveoin-1 (a competitive inhibitor of HO-1) was also increased by 10.9% at 16 hours and by 12.3% by 24 hours. On the other hand, TGase-2 expression was reduced by 5.3% at 16 hours, but the expressions of LC3 and p63 increased by 4.7% and 7.4%, respectively, at 24 hours (Fig. 3B1 and B2).

Although 4HR appeared to induce only low levels of stress in RAW 264.7 cells, as indicated by decreases in the expressions of NFkB signaling proteins, it also induced slight increases in the expressions of cellular adaptation-related proteins. These observations suggest NFkB signaling adversely affected cells, and that the observed atypical expressions of NFkB signaling molecules were induced by 4HR.

### Effects of 4HR on the expressions of antioxidant-related proteins in RAW 264.7 cells

4HR induced a marked increase in SOD-1 (superoxide dismutase-1, 11.2%) at 8 hours and gradual increases in GST-1 (14.3%), PGC-1α (peroxisome proliferator-activated receptor gamma coactivator 1α, 11.6%), HO-1 (heme oxygenase-1, 11.4%), NOS-1 (nitric oxide synthase 1, 6.6%), sodium-dependent vitamin C transporter (SVCT2, 7.2%), and LC3 (4.7%) protein levels at 24 hours versus non-treated controls. On the other hand, the expressions of HSP-27, -70, -90, and NRF-2 (nuclear respiratory factor-2) changed by <±5% (Fig. 3C1 and C2). Thus, 4HR conspicuously activated antioxidant-related proteins, which suggests 4HR might be involved in free radical production in the cytoplasm.

### Effects of 4HR on the expressions of inflammation-related proteins in RAW 264.7 cells

**Figure 4:**
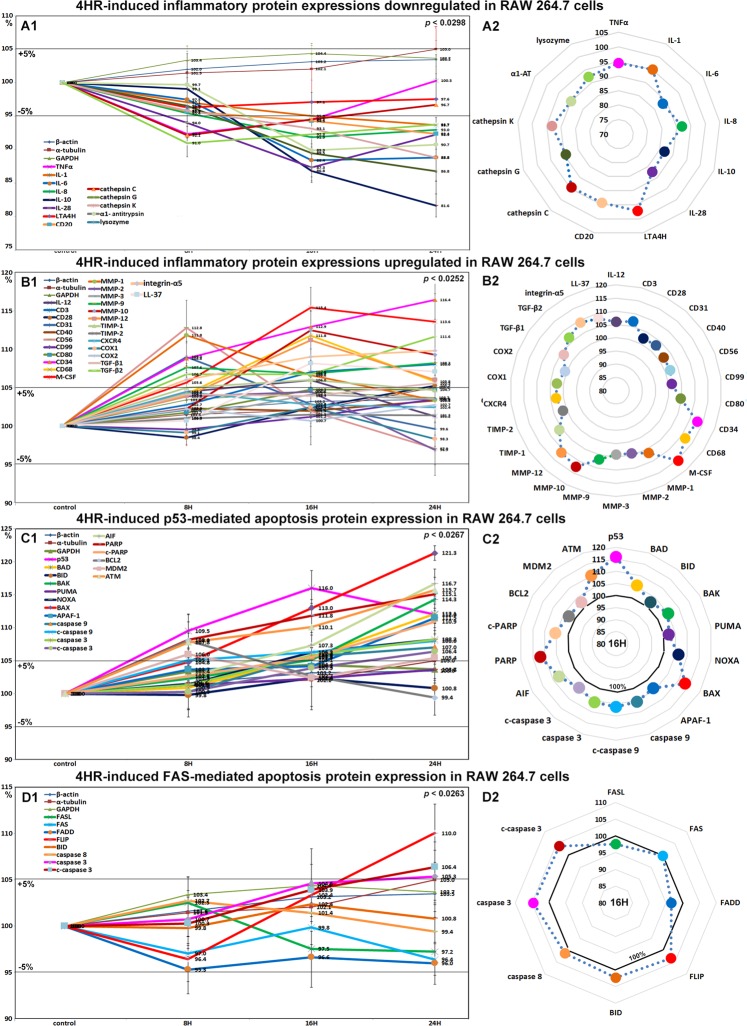
Expressions of downregulated (**A1** and **A2**) and upregulated (**B1** or **B2**) inflammatory proteins, p53-mediated (**C1** and **C2**) and FAS-mediated (**D1** or **D2**) apoptosis-related proteins by 4HR in RAW 264.7 cells, as determined by IP-HPLC. Line graphs, (**A1**, **B1**, **C1**, and **D1**) showed protein expressional changes in the same scale (%) with respect to culture time (8, 16, or 24 hours), whereas circular graphs (**A2**, **B2**, **C2**, and **D2**) showed protein expression levels after 16 hours of culture.

The expressions of inflammation-related proteins were down- or up-regulated by 4HR. Proteins downregulated by 4HR were; TNFα (7.6%), NFkB (5.6%), IL-1 (6.3%), IL-6 (11.6%), IL-8 (8.1%), IL-10 (18.4%), IL-28 (12.7%), cathepsin C (7.9%), cathepsin G (13.2%), cathepsin K (11.2%), lysozyme (9%), LTA4H (4.7%), and CD20 (7.6%) (Fig. 4A1 and A2), and those upregulated were IL-12 (6%), CD3 (8%), CD28 (5.2%), CD31 (9%), CD34 (16.4%), CD40 (3.3%), CD56 (12.8%), CD68 (11.8%), CD80 (4.7%), CD99 (3.5%), LL-37 (8.1%), M-CSF (15.4%), MMP-1 (11.8%), MMP-2 (4.4%), MMP-3 (4.1%), MMP-9 (8.2%), MMP-10 (12.5%), MMP-12 (11.1%), TIMP-1 (6%), TIMP-2 (1.5%), CXCR4 (4.4%), integrin-α5 (9.8%), COX1 (2.4%), COX2 (2.7%), TGF-β1 (5.5%), and TGF-β2 (11.6%) (Fig. 4B1 and B2).

Most of the proteins downregulated by 4HR were inflammatory cytokines, which suggested 4HR might inhibit inflammatory signaling. However, 4HR only mildly affected the expressions of COX1 (a constitutive enzyme for homeostasis), and COX2 (an inducer of inflammation). Therefore, we considered 4HR might have a potent anti-inflammatory effect on RAW 264.7 cells by suppressing TNFα signaling but not COX1 or COX2 signaling. On the other hand, the majority of proteins upregulated by 4HR were related to cellular immunity, innate immunity, and matrix regeneration. The observed anti-inflammatory effect of 4HR might appear to contradict with its immune stimulatory effect in RAW 264.7 cells. However, cells treated with 4HR showed reduced phagocytosis activity due to the downregulations of lysozyme, cathepsin-C, -G, and -K, LTA4H, and IL-8, but increased tissue degradation due to the upregulations of MMP-1, -2, -3, -9, -10, and -12 after 16 hours of treatment, and subsequently exhibited activations of cell-mediated immune proteins including CD3, CD28, CD31, CD34, CD40, CD56, CD68, CD80, and CD99.

### Effects of 4HR on the expressions of p53-mediated apoptosis-related proteins in RAW 264.7 cells

4HR upregulated the expressions of p53-mediated apoptosis-related proteins, particularly p53 protein, which was increased by 16% and 12% at 16 and 24 hours, respectively. The expressions of the apoptosis-related proteins, AIF, APAF-1, c-PARP, c-caspase 9 were moderately increased by 11.7%, 11.5%, 10.9%, and 8%, respectively, and the expressions of DNA repair-related proteins, ATM, BRCA1, and BRCA2 were also moderately increased by 15.7%, 13.2%, and 12.5%, respectively. On the other hand, the expression of anti-apoptotic BCL2 increased by 8% at 8 hours but decreased to the non-treated control level at 24 hours (Fig. 4C1 and C2). These results indicate 4HR enhanced p53-mediated apoptosis and activated DNA repair signaling in RAW 264.7 cells. Therefore, it would appear 4HR induced mitochondria-associated apoptosis in cytoplasm and DNA damage in nuclei.

### Effects of 4HR on the expressions of FAS-mediated apoptosis-related proteins in RAW 264.7 cells

RAW 264.7 cells treated with 4HR showed general decreases in the expressions of FAS-mediated apoptosis-related proteins as compared with non-treated controls. The expressions of FASL, FAS, and FADD were reduced by 2.8%, 3.6%, and 4.7%, respectively, but those of FLIP, caspase 8, caspase 3, and c-caspase 3 were increased by 10%, 2.7%, 5.3%, and 6.4%. Although FASL/FAS signaling was slightly downregulated by 4HR, the expressions of executors of apoptosis, that is, caspase 8, caspase 3, and c-caspase 3, were consistently upregulated despite the increase in FLIP expression. On the other hand, the expression of BID (2.4%, a pro-apoptotic Bcl-2 protein which can be cleaved by caspase 8) changed minimally (<±5%) (Fig. 4D1 and D2). These findings suggest 4HR might induce cellular apoptosis via caspase 8/caspase 3 signaling but independently of FASL/FAS/FADD/FLIP and BID signaling.

### Effects of 4HR on the expressions of angiogenesis-related proteins in RAW 264.7 cells

**Figure 5:**
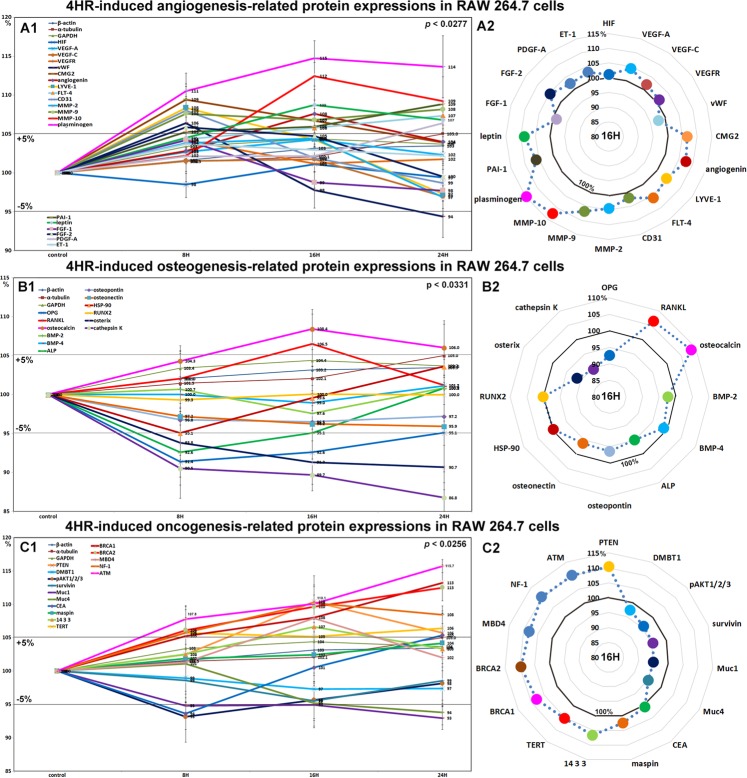
Changes in the expressions of angiogenesis-related proteins (**A1** and **A2**), osteogenesis-related proteins (**B1** or **B2**), and oncogenic proteins (**C1** or **C2**) induced by 4HR in RAW 264.7 cells, as determined by IP-HPLC. Line graphs (**A1**, **B1**, and **C1**) show protein expressional changes in the same scale (%) with respect to culture time (8, 16, or 24 hours), whereas circular graphs (**A2**, **B2**, and **C2**) showed protein expression levels after 16 hours of culture.

RAW 264.7 cells treated with 4HR showed rapid increases in the expressions of angiogenesis-related proteins, as follows, lymphatic vessel endothelial hyaluronan receptor 1 (LYVE-1, 8.4%), vWF (6.4%), capillary morphogenesis protein 2 (CMG2, 9.4%), FGF-2 (5.9%), and CD31 (8%) at 8 hours, and angiogenin (7.6%) and leptin (8.7%) at 16 hours, though the expressions of these 7 proteins subsequently decreased to 106.8–94.4% at 24 hours versus non-treated controls. The expressions of Fms-related tyrosine kinase 4 (FLT-4), MMP-9, PDGF-A, plasminogen, and plasminogen activator inhibitor-1 (PAI-1) were increased by 7.3%, 8.2%, 6.4%, 13.6%, and 8.8% at 16 hours, respectively, but the expressions of VEGF-A, VEGF-C, FGF-1, and ET-1 were increased by <±5%, and the expression of HIF-1α was almost unaffected. In particular, the protein expressions of the extracellular matrix proteases MMP-2, MMP-9, and MMP-10 were all increased by 4.4%, 8.2%, and 12.5%, respectively (Fig. 5A1 and A2).

Angiogenesis-related proteins associated with extracellular matrix, that is, LYVE-1, vWF, capillary morphogenesis protein 2, FGF-2, CD31, angiogenin, leptin, and FLT-4, were upregulated by 4HR, while angiogenic proteins for endothelial cell differentiation, VEGF-A, VEGF-C, and ET-1 were relatively unaffected. The tissue regeneration factors, MMP-2, -9, -10, PDGF-A, plasminogen, and PAI-1, were consistently upregulated by 4HR, indicating 4HR-induced angiogenesis was closely related to active regeneration of extracellular matrix.

### Effects of 4HR on the expressions of osteogenesis-related proteins in RAW 264.7 cells

4HR decreased the expressions of osteogenesis proteins, that is, osteoprotegerin (OPG, 8.6%), alkaline phosphatase (ALP, 7.4%), osterix (9.3%), osteopontin (3.5%), and osteonectin (4.1%), increased the expressions of the osteoclastogenesis-related proteins RANKL (receptor activator of nuclear factor kappa-B ligand) and osteocalcin by 6.5% and 8.4% at 16 hours, respectively, but decreased the expressions of cathepsin K and HSP-90 by 13.2% and 4.9%, respectively. The expressions of BMP-2, BMP-4, and RUNX2 were minimally changed (<±5%) (Fig. 5B1 and B2). These results show 4HR markedly increased osteoclastic differentiation and consistently reduced osteoblastic differentiation in RAW 264.7 cells.

### Effects of 4HR on the expressions of oncogenic proteins in RAW 264.7 cells

RAW 264.7 cells treated with 4HR at 16 hours showed slight increases in the protein expressions of PTEN (phosphatase and tensin homolog, 10.4%), TERT (telomerase reverse transcriptase, 5.2%), and 14-3-3 (6.6%) but slight reductions in the expressions of pAKT1/2/3 (4.4%), mucin 1 (Muc1, 5.1%), Muc4 (4.8%), and survivin (4.5%). The protein expression of carcinoembryonic antigen (CEA) was decreased by 6.4% at 8 hours but increased slightly to a reduction of 5.4% at 24 hours as compared with non-treated controls. On the other hand, the expression of DMBT1 (deleted in malignant brain tumors 1) altered by <±5% (Fig. 5C1 and C2). These concomitant increases in the protein expressions of antioxidant-related proteins and oncogenic proteins suggest roles in latent cellular stress and oncogenesis.

### 4HR adherent assay using acrylamide beads *in vitro*

4HR adherent assays were used to detect 4HR adherent or non-adherent proteins on 4HR coated acrylamide beads as compared with non-coated acrylamide beads. Of the proteins differentially expressed in 4HR-treated RAW 264.7 cells, many proteins (n = 86) were weakly adherent to 4HR coated beads by <±5%, though some proteins (n = 23) were more adherent to 4HR-coated acrylamide beads than non-coated beads by 5.1~18.2%, and the other proteins (n = 26) were less adherent to 4HR-coated acrylamide beads than to non-coated acrylamide beads by −5.2~−17.3% (Table 1).

**Table 1:** Protein adherence to 4HR-coated acrylamide beads as compared with non-coated acrylamide beads.

Range	Number	4HR Adherence of different proteins (%)
10% to 20%	4	TNFα (18.2), PKC (13.4), osteopontin (12.3), GADD45 (10.5)
5% to 10%	19	lysozyme (9.8), OPG (9.7), osteocalcin (9.7), KDM4D (9.5), ET-1 (9.2), PDGF-A (9), pAKT (8.5), Muc1 (7.7), SMAD4 (7.6), E2F-1 (7.5), p-PKC (7.3), MAX (7.3), FLT-4 (7.2), RANKL (7.2), Rb-1 (6.8), p-ERK-1 (5.7), caspase 8 (5.4), ERβ (5.1), NOS-1 (5.1)
−5% to 5%	86	GHRH (5), DMBT1 (5), MMP-2 (4.9), GH (4.7), TGF-β1 (4.5), osterix (4.4), TGase-2 (4.3), eIF5A1 (4.1), MAD1 (4), ERK-1 (3.9), OC (3.9), LC3 (3.9), IL-1 (3.7), VEGF-A (3.6), p38 (3.5), vWF (3.4), HDAC-10 (3.2), COX2 (3.1), PCNA (3), HO-1 (2.8), IL-12 (2.7), eIF2AK3 (2.4), HSP-27 (2), TCF-1 (1.9), Ki-67 (1.7), PLK4 (1.6), mTOR (1.5), NFkB (1.5), DMAP1 (1.4), MMP-1 (1.4), NRAS (1.3), TGF-β2 (1.2), CMG2 (1.2), TGase-2 (1.2), BAD (1), survivin (0.8), caspase 9 (0.7), AMPK (0.6), CD20 (0.2), FLIP (0), MMP-10 (−0.2), DHS (−0.2), α1-antitrypsin (−0.3), VEGF-C (−0.4), HIF-1α (−0.5), lamin A/C (−0.6), SOD-1 (−0.7), TERT (−0.9), HGF (−1.2), angiogenin (−1.2), MPM2 (−1.2), IL-28 (−1.3), osteonectin (−1.4), IKK (−1.7), MMP-3 (−1.8), MDR (−1.8), p21 (−1.9), CEA (−1.9), cathepsin G (−2), FGF-1 (−2.1), Muc4 (−2.1), DOHH (−2.2), RAF-B (−2.2), HSP-90 (−2.2), cathepsin K (−2.3), HER1 (−2.4), HSP-70 (−2.4), JNK1 (−2.5), FADD (−2.6), snail (−2.9), AIF (−3), Histone H1 (−3), BID (−3.3), KRAS (−3.2), p27 (−3.3), 14 3 3 (−3.4), BAX (−3.5), eIF5A2 (−3.6), Rab (−3.6), β-catenin (−3.9), p53 (−3.9), DNMT1 (−4.1), LYVE-1 (−4.3), BCL2 (−4.9), caspase 3 (−4.9), IGF−1 (−4.9)
−5% to −10%	22	APAF-1 (−5.2), PGC-1α (−5.6), FASL (−5.6), Wnt1 (−5.7), p14 (−5.8), SVCT2 (−5.8), FAS (−6), MBD4 (−6.2), HER2 (−6.6), p15/p16 (−6.7), BAK (−7.2), COX1 (−7.7), APC (−7.8), IL-10 (−7.8), IGFIIR (−8.1), cathepsin C (−8.7), cMyc (−8.7), GST-1 (−8.9), PLC-β2 (−9.2), p63 (−9.4), Met (−9.5), CD28 (−9.6)
−10% to −20%	4	IL-6 (−10.2), c-caspase 3 (−10.7), CDK4 (−12), c-caspase 9 (−17.3)
total	135	

**Figure 6:**
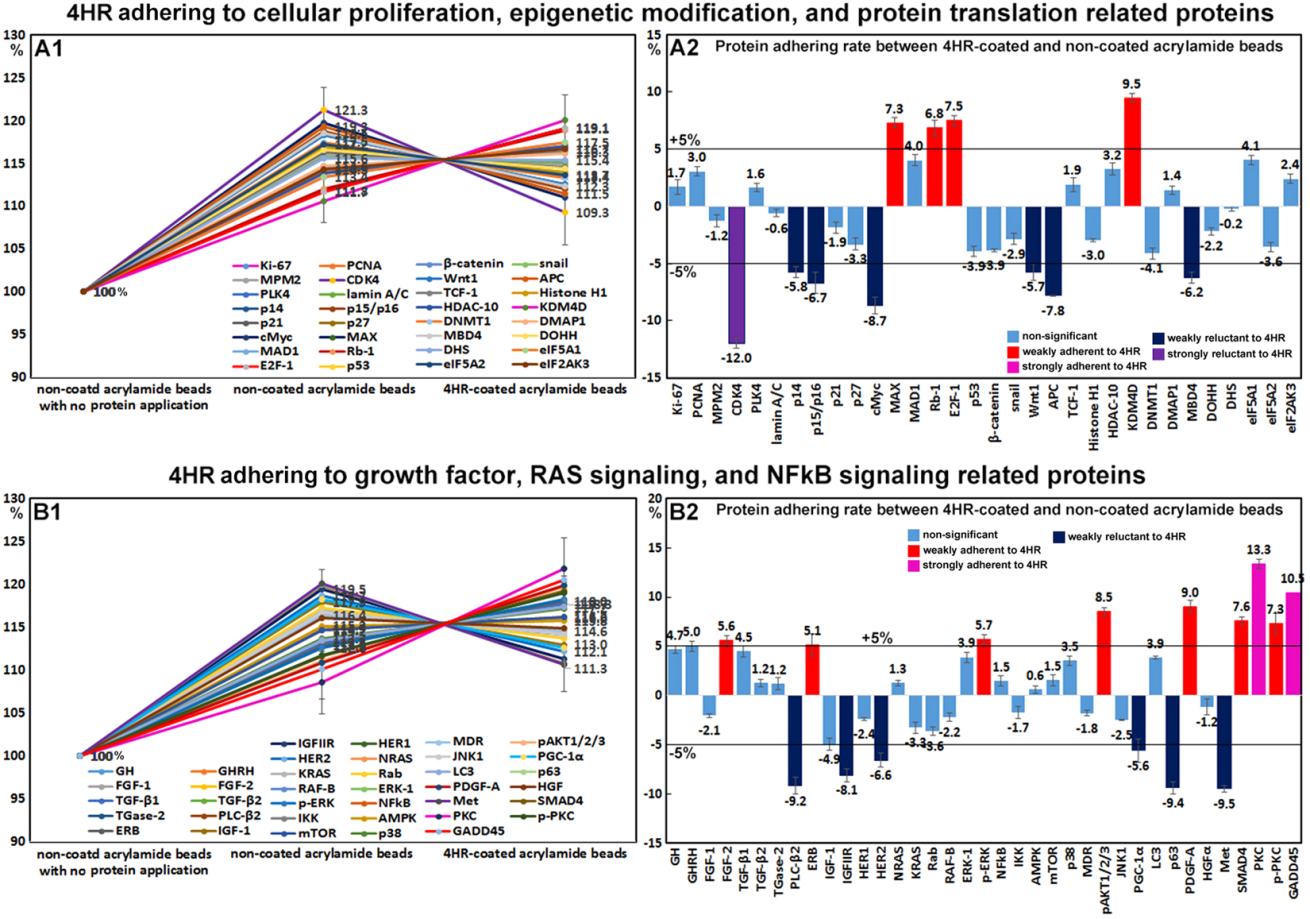
4HR adherence assay results obtained by IP-HPLC. (**A1** and **B1**), line graphs. (**A2** and **B2**), rod graphs. Protein adherences to 4HR-coated acrylamide beads and to non-coated acrylamide beads (positive control) were compared. Line graphs were normalized versus negative control using non-coated beads with no protein application (100%). Rod graphs showing adherence and lack of adherence to 4HR as compared with positive control (100%) as determined by IP-HPLC. A: 4HR adherence to cellular proliferation, epigenetic modification, and protein translation-related proteins. B: 4HR adherence to growth factors, RAS signaling, and NFkB signaling proteins.

**Figure 7:**
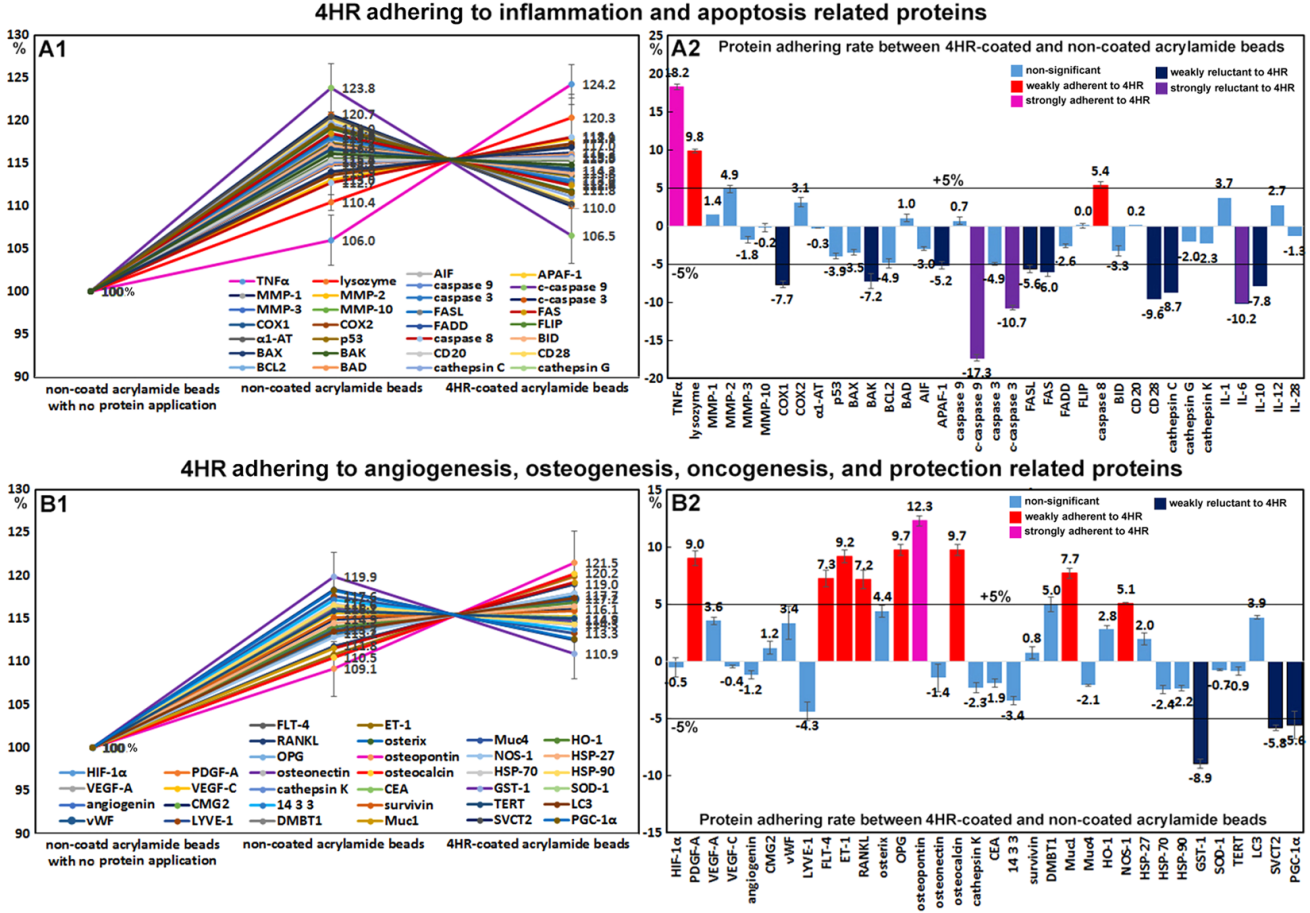
4HR adherence assay results obtained by IP-HPLC. (**A1** and **B1**), line graphs. (**A2** and **B2**), rod graphs. Protein adherences to 4HR-coated acrylamide beads and non-coated acrylamide beads were compared. Line graphs were normalized versus negative control using non-coated beads with no protein application (100%). Rod graphs showing adherence and lack of adherence to 4HR as compared with positive control (100%) as determined by IP-HPLC. A: 4HR adherence to inflammation and apoptosis-related proteins. B: 4HR adherence to angiogenesis, osteogenesis, oncogenesis, and cell protection-related proteins.

TNFα (18.2%), PKC (13.4%), osteopontin (12.3%), and GADD45 (10.5%) were strongly adherent to 4HR-coated beads, and lysozyme (9.8%), OPG (9.7%), osteocalcin (9.7%), KDM4D (9.5%), ET-1 (9.2%), PDGF-A (9%), pAKT (8.5%), Muc1 (7.7%), SMAD (7.6%), E2F-1 (7.5%), p-PKC (7.3%), MAX (7.3%), FLT-4 (7.2%), RANKL (7.2%), Rb-1 (6.8%), p-ERK-1 (5.7%), caspase 8 (5.4%), ERβ (5.1%), and NOS-1 (5.1%) were weakly adherent. IL-6 (−10.2%), c-caspase 3 (−10.7%), CDK4 (−12%), and c-caspase 9 (−17.3%) were substantially more adherent to non-coated beads than 4HR-coated beads, and APAF-1 (−5.2%), PGC-1α (−5.6%), FASL (−5.6%), Wnt1 (−5.7%), p14 (−5.8%), SVCT2 (−5.8%), FAS (−6%), MBD4 (−6.2%), HER2 (−6.6%), p15/p16 (−6.7%), BAK (−7.2%), COX1 (−7.7%), APC (−7.8%), IL−10 (−7.8%), IGFIIR (−8.1%), cathepsin C (−8.7%), cMyc (−8.7%), GST-1 (−8.9%), PLC-β2 (−9.2%), p63 (−9.4%), Met (−9.5%), and CD28 (−9.6%) were slightly more adherent to non-coated beads (Figs 6 and 7).

**Figure 8:**
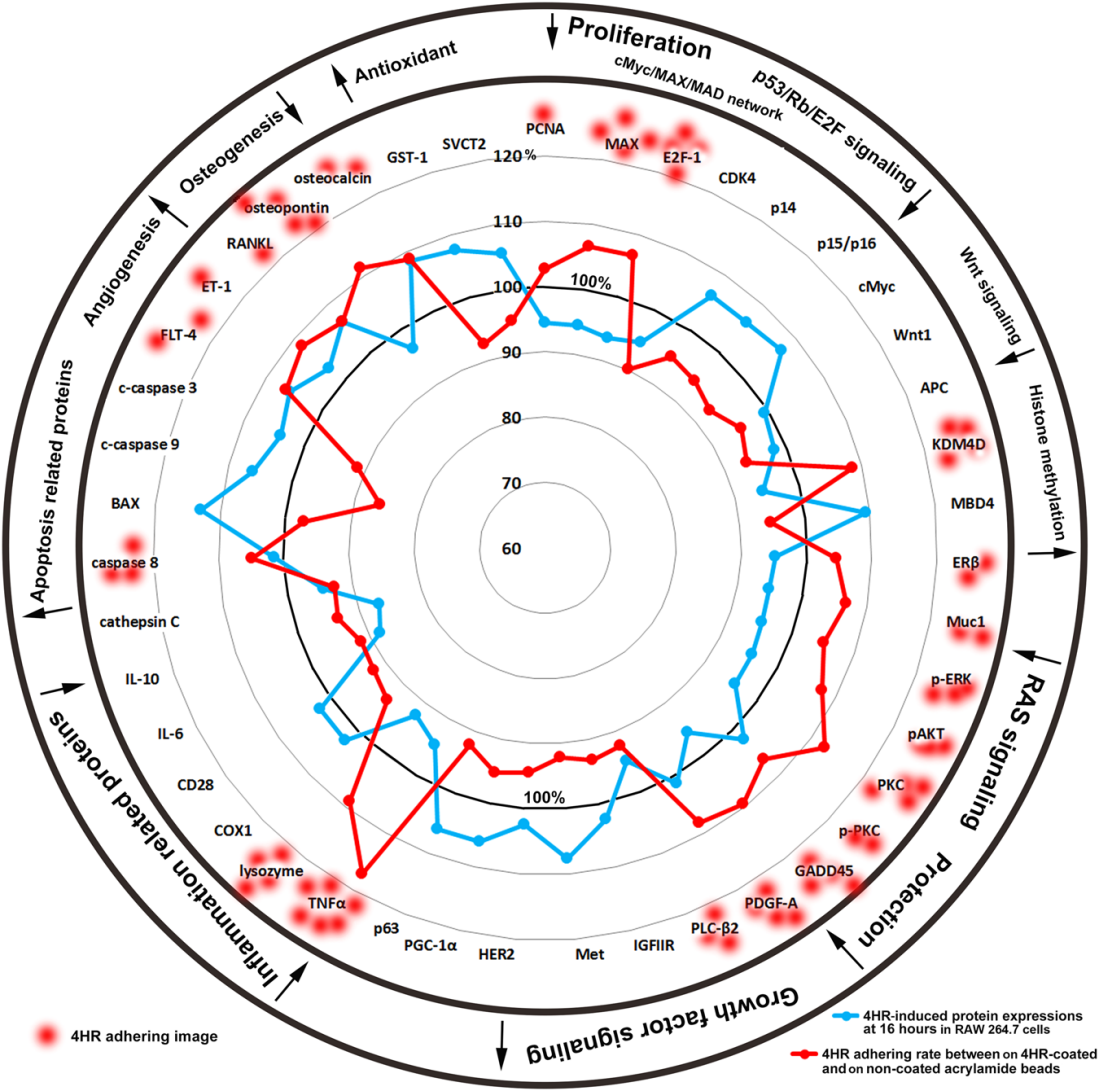
STAR plot representation of 4HR adherence assay results. Many proteins that bound to 4HR were downregulated when RAW 264.7 cells were treated with 4HR, and conversely most proteins that did not bind to 4HR were upregulated by 4HR. Blue line: protein expressional changes (%) in RAW 264.7 cells induced by treatment with 4HR for 16 hours. Red line: 4HR adherence (%).

As determined by IP-HPLC, the expressions of the 4HR adherent proteins, PCNA, MAX, E2F-1, KDM4D, ERβ, Muc1, p-ERK, pAKT, PKC, p-PKC, GADD45, PDGF-A, PLC-β2, TNFα, lysozyme, caspase 8, FLT-4, ET-1, RANKL, osteopontin, and osteocalcin were lower in 4HR-treated RAW 264.7 cells than in non-treated controls, whereas the expressions of the 4HR non-adherent proteins, CDK4, p14, p15/p16, cMyc, Wnt1, MBD4, IGFIIR, Met, HER2, PGC-1α, p63, COX1, CD28, IL-6, IL-10, cathepsin C, BAX, c-caspase 9, c-caspase 3, GST-1, and SVCT2 were higher. These results indicate 4HR interference induces global protein expressions that correspond to the downregulations of cellular proliferation, p53/Rb/E2F, Wnt/β-catenin, and RAS signalings, and cell protection, inflammation, and osteogenesis, but to the upregulations of histone methylation, growth factor signaling, apoptosis, angiogenesis, and antioxidant signaling in RAW 264.7 cells (Fig. 8).

## Discussion

4HR is a phenolic compound with a hexane chain, and as a result is strongly hydrophobic, freely soluble in ether and acetone but only sparingly soluble in water (0.5 mg/mL at 18 °C). Recently, the use of 4HR was extended to local anesthetics, antiseptics, anthelmintics, throat lozenges, skincare products, and anti-aging creams^2^. 4HR has also been shown to exhibit estrogenic activity when used as a food additive^21^ and has been suggested to have anticancer effects in animals by reducing the expression of NFkB^22^.

The present study shows 4HR interacts with proliferation-related proteins, the cMyc/MAX/MAD network, p53/Rb/E2F and Wnt/β-catenin signalings, epigenetic modification, and protein translation, which indicates it has anti-proliferative effects. 4HR was also found to increase the expressions of cellular adaptation, immunity, antioxidant, apoptosis, angiogenesis, and oncogenic-related proteins, which suggests a compensation mechanism to overcome the extensive downregulation of proliferation and growth, epigenetic modification, inflammation, and osteogenesis-related proteins. When RAW 264.7 cells were treated with 4HR, caveolin-1, p63, PGC-1α, p-PKC and LC3 were dominantly upregulated, while PLC-β2, pAKT1/2/3, PI3K, and PKC were consistently downregulated. In terms of 4HR-induced antioxidant signaling, SOD-1, GST-1, HO-1, and NOS-1 were dominantly upregulated, and as regards 4HR-induced angiogenic effects, vWF, LYVE-1, FLT-4, and angiogenin were dominantly upregulated independently of HIF-1α. Finally, concerning oncogenesis, 4HR dominantly upregulated PTEN, ATM, MBD4, BRCA1, BRCA2, TERT, and 14-3-3, but markedly downregulated Muc1, Muc4, pAKT1/2/3, and survivin. Therefore, although 4HR exhibited a silencing effect on cellular proliferation, growth, inflammation, and epigenetic modification, it might also induce a low level of cellular stress in RAW 264.7 cells.

Although RAW 264.7 cells treated with 4HR showed a slight reduction (<10%) in proliferation and growth signaling, 4HR extensively affected RAW 264.7 cells by inactivating epigenetic modification, protein translation, the cMyc/MAX/MAD network, p53/Rb/E2F signaling, Wnt/β-catenin signaling, RAS signaling, and NFkB signaling. Furthermore, our 4HR adherence assay showed 4HR interacted positively or negatively with Ki-67, KDM4D, CDK4, and MAX, which are essential for cellular proliferation and growth.

4HR had a potent anti-inflammatory effect on RAW 264.7 cells by downregulating different inflammation-related proteins, such as TNFα, IL-1, -6, -8, -10, -28, cathepsin-C, -G, -K, lysozyme, α1-antitrypsin, CD20, CD28, and TGF-β1, but upregulated immunity-related proteins, such as IL-12, CD34, CD56, CD68, M-CSF, COX1, COX2, MMP-1, -9, -10, -12, and LL-37. Furthermore, our adherence assay showed 4HR bound to TNFα, lysozyme, IL-1, -12, MMP-2, COX2, and TGF-β1, but not to CD28, cathepsin C, IL-6, IL-10, and COX1. These data suggest that 4HR binding differentially affects the expressions of target proteins essentially required for inflammation and immunity.

Our results show 4HR is biologically active and induces anti-proliferative and anti-inflammatory effects in RAW 264.7 cells, and suggest it may also induce a low level of cellular stress, because it upregulated the expressions of antioxidant and oncogenic-related proteins. The effect of 4HR on cellular stress was also observed to be compensated for by increases in the expressions of cellular adaptation and angiogenesis-related proteins. Furthermore, the protein expressional changes induced by 4HR were usually in the range <±10%, though some RAS signaling proteins (KRAS, NRAS, RAF-B, and JAK2), inflammatory proteins (IL-6, -10, -28, CD-34, M-CSF, MMP-10, and cathepsin G), apoptosis-related proteins (p53, BAX, and caspase 3), and oncogenic proteins (ATM, BRCA1, and BRCA2) exhibited expressional changes of around ±15%. These results indicate that 4HR has only mild effects on RAW 264.7 cells.

4HR slightly increased p53-mediated apoptosis and upregulated antioxidant-related proteins in RAW 264.7 cells, which indicates 4HR induced cellular scavenging under conditions that diminish proliferation and growth. This is in-line with the reported induction of cellular dormancy by 4HR in micro-organisms^23,24^, and suggests 4HR might exhibit synergistic effects if administered in combination with anticancer drugs^22^.

Our 4HR adherence assay showed 4HR up- or down-regulated different proteins in RAW 264.7 cells, and that it positively or negatively interacted with proteins when coated on acrylamide beads. Because 4HR is strongly hydrophobic, it may interact with the hydrophobic domains of proteins^25^. We found 4HR-coated acrylamide beads markedly absorbed TNFα, PKC, osteopontin, and GADD45, and slightly absorbed lysozyme, OPG, osteocalcin, KDM4D, ET-1, PDGF-A, pAKT, Muc1, SMAD4, E2f-1, p-PKC, MAX, FLT-4, RANKL, Rb-1, p-ERK, caspase 8, ERβ, and NOS-1, and absorbed IL-6, c-caspase 3, CDK4, c-caspase 9 less than non-coated acrylamide beads. Although we could not explore molecular interactions affecting protein conformations, 4HR adherence and lack of adherence to proteins tended to be correlated with the negativity or positivity, respectively, of their expressional changes in 4HR-treated RAW 264.7 cells (Fig. 8). These results suggest 4HR influences protein expressions differentially in RAW 264.7 cells, and that these influences are related to adherence.

It has also been reported that 4HR negatively effects TNFα^26^, NFkB^22^, and TGase-2^27^ levels but positively effects lysozyme levels^25,28^. The 4HR adherence assay performed in the present study showed 4HR influenced the levels of many proteins in RAW 264.7 cells, and our adherence results suggest its interaction with proteins is non-specific but depends on the strengths of hydrophobic interactions.

In the present study, 4HR adherence to different proteins was observed to be associated with global protein expressional changes in RAW 264.7 cells. These molecular interactions between 4HR and different proteins differ from those between molecular chaperones and target proteins^29^, and we formed the opinion that observed 4HR-induced protein expressional changes were derived from active molecular interference by 4HR. Molecular interactions between 4HR and proteins are likely to be dynamic and dependent on cell status and type, and therefore, we recommend further investigations to be conducted to elucidate the mechanism responsible for 4HR-induced changes in protein conformations.

In the present study, we explored the effects of 4HR on RAW 264.7 cells by IP-HPLC using 216 antisera. Treated cells showed significant decreases in the expressions of proliferation-related proteins, cMyc/MAX/MAD network, p53/Rb/E2F and Wnt/β-catenin signalings, epigenetic modifications, and protein translation. In addition, 4HR suppressed the expressions of growth factors and proteins associated with RAS signaling, NFkB signaling, inflammation, and osteogenesis to different extents, but elevated the expressions of proteins associated with p53-mediated apoptosis, T-cell immunity, angiogenesis, antioxidant, and oncogenic signaling. The 4HR adherence assay showed TNFα, PKC, osteopontin, and GADD45 were strongly adherent to 4HR-coated beads, whereas IL-6, c-caspase 3, CDK4, and c-caspase 9 were not. Furthermore, the expressions of most proteins that adhered to 4HR were down-regulated by 4HR, whereas non-adherent proteins were up-regulated. These observations suggest the effects of 4HR on protein expressions are related its ability to interact with different proteins, and that its cellular effects are due to characteristic and induced global protein expressional changes in RAW 264.7 cells.

## Materials and Methods

### RAW264.7 cell cultures in the presence of 4HR

RAW 264.7 cells (an immortalized murine macrophage cell line; ATCC, USA) were cultured in Dulbecco’s modified Eagle’s medium (WelGene Inc. Korea) supplemented with 10% (vol/vol) heat-inactivated fetal bovine serum (WelGene Inc. Korea), 100 unit/mL penicillin, 100 μg/mL streptomycin, and 250 ng/mL amphotericin B (WelGene Inc. Korea), in 5% CO_2_ at 37.5 °C. Cells were cultured in antigen free media in order to detect native protein expressional changes induced by 4HR.

About 70% confluent RAW 264.7 cells grown on Petri dish surfaces were treated with 10 µg/mL 4HR (safe single dose in dogs 100–300 mg/kg according to WHO food additives Series 35, 835) for 8, 16, or 24 hours; control cells were treated with 1 mL of normal saline. Cultured cells were harvested with protein lysis buffer (PRO-PREP^TM^, iNtRON Biotechnology INC, Korea), and immediately preserved at −70 °C until required.

### 4HR adherence assay using acrylamide beads *in vitro*

In order to detect 4HR adherent proteins, acrylamide beads were coated with 4HR by adding 10 mL of ethanol saturated with 4HR to 1 L of normal saline solution containing acrylamide beads (30 mL, Sephacryl^TM^ S-300, Amersham Pharmacia Biotech. Sweden) in chromatography columns (Bio-Rad, USA). Beads were coated with 4HR by stirring the mixture and became brown in color. 4HR coated acrylamide beads were washed with 1.5 M NaCl solution, incubated with total protein extract from RAW 264.7 cells in 50 mM Tris buffer (pH 7.5) for 1 hour, and washed with normal saline solution three times. They were then incubated with 1.5 M NaCl solution for 10 minutes to elute 4HR adherent proteins. Eluted proteins were analyzed by IP-HPLC as described above using 135 antisera. Results were compared with those obtained for non-coated acrylamide beads (Supplemental Data 1 and Fig. 1).

### Immunoprecipitation high-performance liquid chromatography (IP-HPLC)

Protein extracts (100 μg) were subjected to immunoprecipitation using a protein A/G agarose column (Amicogen, Korea). Protein A/G agarose columns were separately pre-incubated with 1 μg of 216 different antisera for; growth and proliferation-related proteins (n = 10), cMyc/MAX/MAD signaling proteins (n = 3), p53/Rb/E2F signaling proteins (n = 5), epigenetic modification-related proteins (n = 6), protein translation-related proteins (n = 5), RAS signaling proteins (n = 17), growth factor-related proteins (n = 16), NFkB signaling proteins (n = 12), cellular protection-related proteins (n = 15), upregulated inflammatory proteins (n = 26), downregulated inflammatory proteins (n = 13), p53-mediated apoptosis-related proteins (n = 17), FAS-mediated apoptosis-related proteins (n = 8), angiogenesis-related proteins (n = 20), osteogenesis-related proteins (n = 12), antioxidant-related proteins (n = 8), oncogenic proteins (n = 15), and control housekeeping proteins (n = 3) (Table 2).

**Table 2:** Antibodies used in the study.

Signaling proteins	No.	Antibodies
Cellular proliferation	10	Ki-67^*^, PCNA^*^, CDK4^*^, PLK4^*^, lamin A/C, MPM2^*^, p14^*^, p16^*^, p21^*^, p27^*^
cMyc/MAX/MAD signaling	3	cMyc^*^, MAX^*^, MAD^*^
p53/Rb/E2F signaling	5 (2)	p53, Rb-1^#^, E2F-1^*^, (p21, CDK4)
Wnt/β-catenin signaling	5	Wnt1^*^, β-catenin^*^, APC^*^, snail^*^, TCF-1^*^
Epigenetic modification	6	DMAP1^*^, histone H1^*^, KDM4D^$^, HDAC-10^$^, MBD4^*^, DNMT1^*^
Protein translation signaling	5	DOHH^*^, DHS^*^, elF5A-1^$^, elF5A-2^$^, eIF2AK3^*^
RAS signaling	17	NRAS^$^, KRAS^$^, STAT3^*^, SOS-1^*^, SOS-2^*^, RAF-B^*^, JAK2^$^, JNK-1^*^, ERK1^*^, Rab^*^, p-ERK^$^, AP-1^@^, SP-1^@^, SP-3^@^, AMPK^@^, (PKC^*^, p-PKC^@^)
Growth factor signaling	16	FGF-1^*^, FGF-2^*^, HGF^*^, TGF-β1^#^, TGF-β2, SMAD4^*^, PDGF-A^*^, IGF-1^*^, IGFIIR^*^, GH^*^, GHRH^*^, HER1^*^, HER2^*^, ERβ^*^, insulin^@^, Met^*^
NFkB signaling	12 (3)	NFkB^*^, IKK^*^, GADD45^*^, MDR, mTOR^@^, p38^*^, p-p38^*^, NRF-2^*^, IL-1^*^, (ERK1^*^, p-ERK^*^, AMPK)
Upregulated inflammatory proteins	26 (2)	IL-12^*^, CD3^*^, CD28^*^, CD31^*^, CD34^*^, CD40^*^, CD56^*^, CD68^*^, CD80^*^, CD99^*^, LL-37^*^, M-CSF^*^, MMP-1^$^, -2^$^, -3^$^, -9^$^, -10^$^, -12^$^, TIMP-1^&^, TIMP-2^&^, CXCR4^*^, COX1^*^, COX2^*^, integrin- α5^*^, (TGF- β1^#^, TGF- β2^*^)
Downregulated inflammatory proteins	13 (1)	TNFα^@^, IL-1^*^, IL-6^*^, IL-8^*^, IL-10^*^, IL-28^*^, LTA4H^&^, α1- antitrypsin ^&^, lysozyme^*^, CD20^$^, cathepsin C^*^, cathepsin G^*^, cathepsin K^*^,
Cellular protection-related	15 (2)	LC3, PLC- β2, PI3K, PKC^*^, p-PKC^*^, FAK^*^, caveolin-1^*^, PGC-1α^*^, HSP-27^*^, HSP-70^*^, HSP-90^*^, TGase 2^$^, p63^$^, (pAKT1/2/3^*^, HO-1)
Antioxidant-related	8 (3)	HO-1^*^, SOD-1^*^, GST-1^*^, SVCT2^&^, NOS-1^$^, (PGC-1α ^$^, LC3^*^, NRF-2)
p53-mediated cellular apoptosis	17 (1)	(p53^*^), PUMA^*^, NOXA^*^, MDM2^*^, BCL2^*^, BAX^*^, BAD^*^, BAK^*^, BID^*^, AIF^*^, APAF-1^*^, caspase 9^*^, c-caspase 9^*^, caspase 3^*^, c-caspase 3^*^, PARP^*^, c-PARP^*^
FAS-mediated cellular apoptosis	8 (3)	FASL^*^, FAS^*^, FADD^*^, FLIP^*^, caspase 8^*^, (BID^*^, caspase 3^*^, c-caspase 3^*^)
Oncogenic proteins	15 (2)	PTEN^&^, MUC1, MUC4, maspin^*^, BRCA1^&^, BRCA2^&^, NF-1^*^, ATM^*^, CEA^$^, 14-3-3^*^, survivin^@^, DMBT1^*^, TERT^*^, (pAKT1/2/3^*^, MBD4)
Angiogenesis-related proteins	20 (7)	HIF^&^, VEGF-A^*^, VEGF-C^*^, angiogenin^$^, LYVE-1^*^, CMG2^$^, vWF^$^, FLT-4^$^, ET-1^*^, PAI-1^*^, VEGFR^*00^, plasminogen^*^, leptin^*^, (CD31, MMP-2, MMP-9, MMP-10, FGF-1, FGF-2, PDGF-A)
Osteogenesis-related proteins	12 (2)	OPG^*^, RANKL^*^, BMP-2^*^, BMP-4^*^, ALP^*^, osteocalcin^*^, osteopontin^*^, osteonectin^*^, RUNX2^*^, osterix^*^, (HSP-90, cathepsin K)
Control housekeeping proteins	3	α-tubulin^*^, β-actin^*^, GAPDH^*^
Total	216 (28)	

*Santa Cruz Biotechnology, USA; ^#^DAKO, Denmark; ^$^Neomarkers, CA, USA; ^@^ZYMED, CA, USA; ^&^Abcam, Cambridge, UK; the number of antibodies overlapped; ().

Abbreviations: AMPK; AMP-activated protein kinase, pAKT; v-akt murine thymoma viral oncogene homolog, p-Akt1/2/3 phosphorylated (p-Akt, Thr 308), APAF-1; apoptotic protease-activating factor 1, AP-1; activating protein-1, BAD; BCL2 associated death promoter, BAK; BCL2 antagonist/killer, BAX; BCL2 associated X, BCL-2; B-cell leukemia/lymphoma-2, BID; BH3 interacting-domain death agonist, c-caspase 3; cleaved-caspase 3, CD3; cluster of differentiation 3, CDK4; cyclin dependent kinase 4, CEA; carcinoembryonic antigen, CMG2: capillary morphogenesis protein 2, COX-1; cyclooxygenase-2, COX-2; cyclooxygenase-2, c-PARP; cleaved- PARP (poly-ADP ribose polymerase), DMAP1; DNA methyltransferase 1 associated protein, DMBT1; deleted in malignant brain tumors 1, DOHH; deoxyhypusine hydroxylase, DHS; deoxyhypusine synthase, E2F-1; transcription factor, eIF2AK3 (PERK); eukaryotic translation initiation factor 2 (protein kinase R (PKR)-like endoplasmic reticulum kinase), elF5A-1; eukaryotic translation initiation factor 5A-1, elF5A-2; eukaryotic translation initiation factor 5A-2, ERβ; estrogen receptor beta, ERK; extracellular signal-regulated protein kinases, ET-1: endothelin-1, FAS; CD95/Apo1, FASL; FAS ligand, FADD; FAS associated via death domain, FGF-1; fibroblast growth factor-1, FLIP; FLICE-like inhibitory protein, FLT-4; Fms-related tyrosine kinase 4, GADD45; growth arrest and DNA-damage-inducible 45, GAPDH; glyceraldehyde 3-phosphate dehydrogenase, GH; growth hormone, GHRH; growth hormone-releasing hormone, GST-1; glutathione S-transferase ω 1, HDAC-10; histone deacetylase 10, HIF-1α: hypoxia inducible factor-1α, HO-1; heme oxygenase 1, HER1; human epidermal growth factor receptor 1, HGFα; hepatocyte growth factor α, HSP-70; heat shock protein-70, IKK; ikappaB kinase, IGF-1; insulin-like growth factor 1, IGFIIR; insulin-like growth factor 2 receptor, IgK; immunoglobulin kappa (light chain), IL-1; interleukin-1, KDM4D; Lysine-specific demethylase 4D, JNK-1; Jun N-terminal protein kinase, KRAS; V-Ki-ras2 Kirsten rat sarcoma viral oncogene homolog, LC3; microtubule-associated protein 1 A/1B-light chain 3, LYVE-1: lymphatic vessel endothelial hyaluronan receptor 1, MAX; myc-associated factor X, MBD4; methyl-CpG-binding domain protein 4, M-CSF; macrophage colony-stimulating factor, MDM2; mouse double minute 2 homolog, MDR; multiple drug resistance, MMP-1; matrix metalloprotease-1, MPM2; mitotic protein monoclonal 2, mTOR; mammalian target of rapamycin, cMyc; V-myc myelocytomatosis viral oncogene homolog, NFkB; nuclear factor kappa-light-chain-enhancer of activated B cells, NOS-1; nitric oxide synthase 1, NRAS; neuroblastoma RAS Viral Oncogene homolog, NRF2; nuclear factor (erythroid-derived)-like 2, p14, p16, p21, p27, p38, PAI-1; plasminogen activator inhibitor-1, PARP; poly-ADP ribose polymerase, PCNA; proliferating cell nuclear antigen, PDGF-A: platelet-derived growth factor-A, PLC-β2; 1-phosphatidylinositol-4,5-bisphosphate phosphodiesterse β-2, PI3K; phosphatidylinositol-3-kinase, PLK4; polo like kinase 4 or serine/threonine-protein kinase, PKC; protein kinase C, p-p38; phosphor-p38, PTEN; phosphatase and tensin homolog, RANKL; receptor activator of nuclear factor kappa-B ligand, Rb-1; retinoblastoma-1, RUNX2; Runt-related transcription factor-2, SMAD4; mothers against decapentaplegic, drosophila homolog 4, SOD-1; superoxide dismutase-1, SP-1; specificity protein 1, STAT3; signal transducer and activator of transcription-3, TGF-β1; transforming growth factor-β1, TERT; human telomerase reverse transcriptase, TNFα; tumor necrosis factor-α, β-actin, 14-3-3, VEGF vascular endothelial growth factor, VEGFR2: vascular endothelial growth factor receptor 2, p-VEGFR2: vascular endothelial growth factor receptor 2 (Y951), vWF: von Willebrand factor.

Briefly, protein samples were mixed with 5 mL of binding buffer (150 mM NaCl, 10 mM Tris pH 7.4, 1 mM EDTA, 1 mM EGTA, 0.2 mM sodium vanadate, 0.2 mM PMSF and 0.5% NP-40) and incubated in protein A/G agarose (Amicogen, Korea) columns at 4 °C for 1 hour (columns were placed on a rotating stirrer during incubation). After washing each column with sufficient phosphate buffered saline solution, target proteins were eluted with 150 μL of IgG elution buffer (Pierce, USA). Immunoprecipitated proteins were analyzed using a HPLC unit (1100 series, Agilent, USA) equipped with a reverse phase column and a micro-analytical detector system (SG Highteco, Korea). Elution was performed using 0.15 M NaCl/20% acetonitrile solution at 0.4 mL/min for 30 min, and proteins were detected by UV spectroscopy at 280 nm. Control and experimental samples were run sequentially to allow comparisons^12,30,31^. For IP-HPLC, whole protein peak areas (mAU*s) were calculated after subtracting negative control antibody peak areas, and the square roots of protein peak areas were calculated to normalize concentrations (Supplementary Fig. 2). Protein percentages in total proteins in experimental and control groups were plotted. Analyses were repeated two to six times to achieve mean standard deviations of ≤±5%. Results were analyzed using the Chi-squared test.

The expressions of housekeeping proteins, that is, *β*-actin, *α*-tubulin, and glyceraldehyde 3-phosphate dehydrogenase (GAPDH) were used as internal controls. Expressional changes of housekeeping proteins were adjusted to <±5% using a proportional basal line algorithm. To describe protein expressional changes, we tentatively defined a ≤±5% change as minimal, ±5–10% as slight, ±10–20% as meaningful, and a ≥±20% change as marked.

### Statistical analysis

Proportional data (%) of experimental and control groups were plotted, and the analysis was repeated two to six times (until the mean and standard deviations were ≤±5%). The results were analyzed using the Chi-squared test. The expression of control housekeeping proteins, that is, *β*-actin, *α*-tubulin, and glyceraldehyde 3-phosphate dehydrogenase (GAPDH), were relatively unchanged (≤5%) after 8, 16, or 24 hours of 4HR treatment.

## Supplementary information

Effects of 4-Hexylresorcinol on Protein Expressions in RAW 264.7 Cells as Determined by Immunoprecipitation High Performance Liquid Chromatography

Dataset 1

Dataset 2
