# Supplementary material for: Effects of 4-Hexylresorcinol on Protein Expressions in RAW 264.7 Cells as Determined by Immunoprecipitation High Performance Liquid Chromatography
Source: Sci Rep. 2019 Mar 4;9:3379. doi: 10.1038/s41598-019-38946-4 (PMC6399215; doi:10.1038/s41598-019-38946-4)
Supplement: Supplementary file 1 — Effects of 4-Hexylresorcinol on Protein Expressions in RAW 264.7 Cells as Determined by Immunoprecipitation High Performance Liquid Chromatography [file 41598_2019_38946_MOESM1_ESM.docx]

**Effects of 4-Hexylresorcinol on Protein Expressions in RAW 264.7 Cells as Determined by Immunoprecipitation High Performance Liquid Chromatography**

Min Keun Kim^1#^, Cheol Soo Yoon^2#^, Seong Gon Kim^1^, Young Wook Park^1^, Sang Shin Lee^2^, Suk Keun Lee^2^*

^1^Department of Oral and Maxillofacial Surgery, College of Dentistry, Gangneung-Wonju National University, and Institute of Oral Science, Gangneung, Korea. ^2^Department of Oral Pathology, College of Dentistry, Gangneung-Wonju National University, and Institute of Oral Science, Gangneung, Korea. Correspondence and requests for materials should be addressed to S.K.L. (email: sukkeunlee@hanmail.net)

**^#^** Min Keun Kim and Cheol Soo Yoon: Evenly contributed for this paper.
