# Supplementary material for: Effects of 4-Hexylresorcinol on Protein Expressions in RAW 264.7 Cells as Determined by Immunoprecipitation High Performance Liquid Chromatography
Source: Sci Rep. 2019 Mar 4;9:3379. doi: 10.1038/s41598-019-38946-4 (PMC6399215; doi:10.1038/s41598-019-38946-4)
Supplement: Supplementary file 3 — Dataset 2 [file 41598_2019_38946_MOESM3_ESM.docx]

**Effects of 4-Hexylresorcinol on Protein Expressions in RAW 264.7 Cells as Determined by Immunoprecipitation High Performance Liquid Chromatography**

Min Keun Kim, Cheol Soo Yoon, Seong Gon Kim, Young Wook Park, Sang Shin Lee, Suk Keun Lee


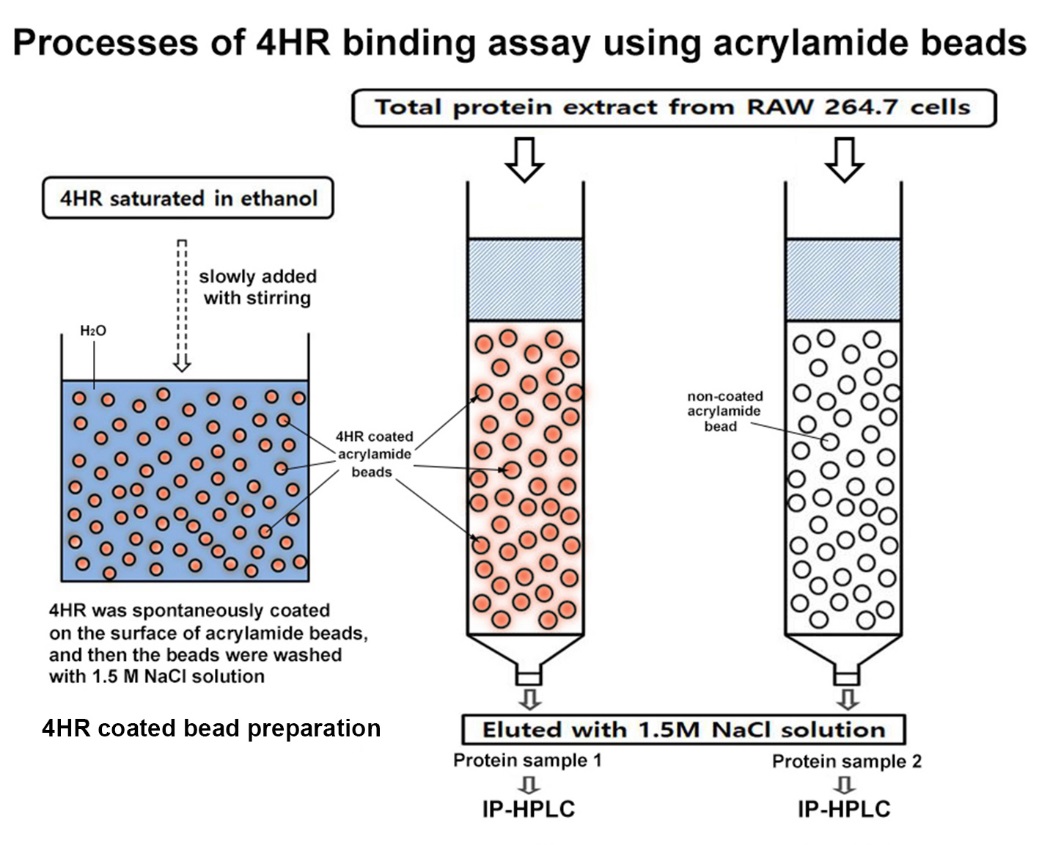


**Supplementary Figure 1.** 4HR adherence assay using 4HR-coated acrylamide beads. Acrylamide beads were coated with 4HR by adding 10 mL of ethanol saturated with 4HR to 1 L of normal saline solution containing acrylamide beads (30 mL, Sephacryl^TM^ S-300, Amersham Pharmacia Biotech. Sweden). Protein extracts of RAW 264.7 cells were loaded into 4HR coated or non-coated acrylamide bead columns for one hour and then columns were eluted with 1.5 M NaCl solution. Eluted proteins were analyzed by IP-HPLC.


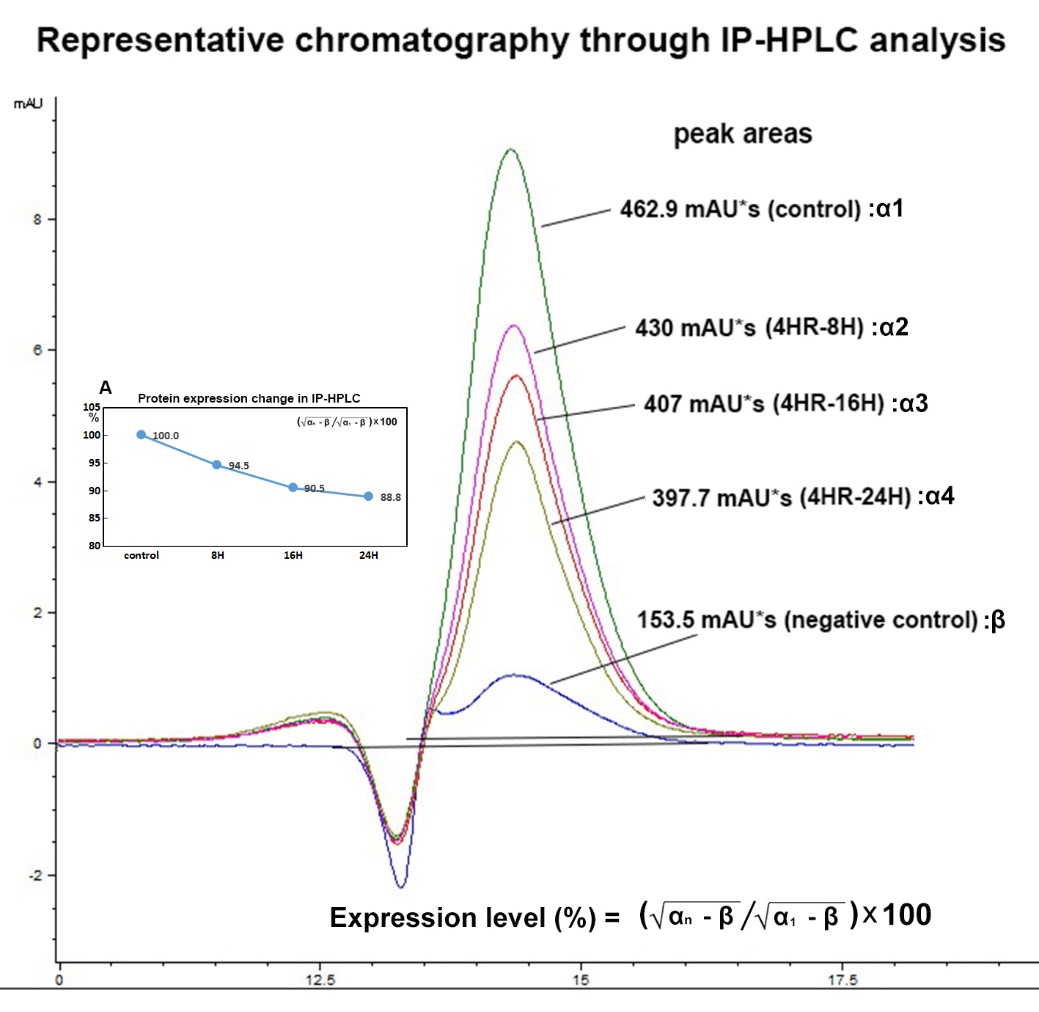


**Supplementary Figure 2.** A representative example of IP-HPLC results for IL-10 expression. Control peak area were subtracted from protein peak areas, and protein expression levels (%) versus the positive control were calculated using the equation ($\sqrt{\alpha n- \beta}$ / $\sqrt{\alpha1- \beta}$ ) x 100 (α: experimental peak area (mAU*s), β: negative control peak area (mAU*s), n: experimental number). Plotted results showed linear decreases of IL-10 expression at 8, 16 24 hours after 4HR treatment (A).
